# Intubation of the Larynx

**Published:** 1892-02-13

**Authors:** 


### LARYNGOLOGY.

### INTUBATION OF THE LARYNX.

It must be very difficult for those who are not practically acquainted with intubation and tracheotomy to weigh the evidence that is brought forward in support of one or the other method of treating stenosis of the larynx. That opinion is still divided, even amongst those who may be regarded as authorities on these subjects, is shown by an examination of some of the recent literature on the subject. Dr. Egidi,* of Rome, has performed the operation in 30 cases, of which 27 were in children and three in adults. Of the children, 21 were cases of primary or simple croup, two of croup consecutive to diphtheria of the fauces, three of croup following measles, one of stenosis following the use of a tracheotomy tube. The youngest child was ten months old, 17 were between one and three years, and nine between three and five years. Omitting the case of stenosis after tracheotomy, of the remaining 26 cases, four recovered, all of primary croup. Introduction of the tube in Egidi's cases was generally easily performed, though in his first case the tube was only inserted after seven attempts. It happened in two cases that membrane was pushed down before the tube, which, on removal of the tube, was coughed up. No great difficulty was experienced in the feeding of any of the children. Coughing up of the tube soon after introduction happened three times, and on the reintroduction of a larger size no further trouble occurred. The tube was worn for periods varying from 22 hours to 13 days in the successful cases. Extraction of the tube was done with the extractor in four cases, but Egidi considers the procedure so difficult that he prefers to leave the thread in situ when possible. In only one case was an erosion from pressure of the tube found post-mortem. The tube had been in place four days, and the erosion was situated in the anterior wall of the trachea, opposite the lower end of the tube. In a paper by Dr. Julius Schwalbe,† of Berlin, reference is first made to the "dreadfully small" percentage of successes after tracheotomy in children with laryngeal diphtheria, and to the difficulties and dangers of the operation, even in the most skilful hands. Tubage of the larynx was tried in thirteen cases, but, for various reasons, three cases had to be excluded from further consideration. Of these ten cases, all suffering from primary diphtheria, nine died and only one recovered. Of the nine fatal cases, one was benefited, viz., a child 3¼ years old, who, in the act of vomiting, expelled the tube, after wearing it twenty-four hours. She died subsequently, but did not suffer from further dyspnœa. Of the remaining eight fatal cases, one child was under one year, four children under two years, and three over two years. Death occurred twice on the second day, once on the third day, twice on the fourth day, once on the seventh day, once on the ninth day, once on the tenth day, and once on the fifteenth day. The cause of death was pneumonia in five cases, purulent bronchitis in two cases; one death was due to fibrinous bronchitis, and one to capillary bronchitis (post-mortem refused). The only case which survived was a boy aged eight years; in this case tubage was undertaken earlier than tracheotomy is usually performed. As tested by the results of these cases, the author thinks that intubation is not likely to improve the statistics of diphtheria. The number of successful tracheotomies averages form 30 to 35 per cent. per annum. Whether better results would have been obtained by tracheotomising the foregoing cases seems doubtful, especially when the ages are taken into consideration, five of the children being under two years; the average results of tracheotomy at that age yield not more than 10 per cent. of recoveries. Among other disadvantages the author refers to the possibility of the tube being brought up, either in consequence of vomiting or of a severe cough; if no one capable of replacing the tube, or of performing tracheotomy is present, fatal consequences may rapidly follow. In one case he found a large quantity of membrane in the trachea that could not possibly have been expectorated through the tube. Reference is made to the displacement of membrane while the tube is being introduced, and to the dangers of this, Dr. Urban, of Leipzig, having recorded 12 cases. A further danger consists in pressure sores. In nine cases examined post-mortem, there were five instances of pressure sores, partly in the trachea, partly in the larynx. Other authors have recorded a similar experience. Thus Widerhofer found sores in nine cases out of 13, in four of which the tube had not been worn more than 24 hours. Schwalbe thinks the difficulties and the dangers of intubation outweigh the advantages, and agrees with Urban, of Leipzig, that it is better to trust to tracheotomy.

When the subject of intubation was discussed at the Berlin Medical Society, Dr. A. Rosenberg * took exception to some of the statements and conclusions of Dr. Schwalbe, to which we have referred. He said the relative merits of intubation and of tracheotomy had not been equally well stated. It was well known that erysipelas or wound diphtheria, or reinfection through the wound, might take place after the latter operation. Granulations within the larynx occasionally followed tracheotomy; sometimes decubitus from pressure of the tube and dangerous hæmorrhage might also ensue. He allowed that certain difficulties in feeding intubated children occurred, but the majority of operators had not found them insuperable. Schwalbe, when referring to these difficulties, had not stated whether he had followed Casselberry's advice to let the head be placed very low—a position which facilitated swallowing after intubation. When the tube was coughed out it was frequently followed by a quantity of membrane. If a membrane did not come up it should be got up with forceps, as was done after tracheotomy. Cough was often due to the thread attached to the tube passing over instead of to one side of the epiglottis in cases where the tube itself was otherwise suitable and well-fitting. The blocking up of the tube with mucus and the insufficient aeration were said to cause atelectasis and subsequent pneumonia. But Ranke's statistics showed little or no difference in the relative frequency of pneumonia after tracheotomy and after intubation. If much disease were present in the lungs, intubation was contra-indicated. As to the frequency of decubitus, though it could not always be prevented, it was an

* *Brit. Med. Journ.*, July 25th, 1891; † *ibid* April 25th, 1891.

* *British Med. Jour.*, July 11th, 1891.

uncommon occurrence to any dangerous extent. Much depended on the dexterity of the operator. It was necessary also to have proper instruments; they should be made exactly on O'Dwyer's model. Statistics on this question would only be of any value when dealing with very large numbers of cases, for then the fallacies (which were unavoidable) were reduced to their lowest average. Different epidemics of diphtheria gave different results, by whatever method treated. While upholding intubation, he did not for a moment imagine that it would displace tracheotomy. In other than the acute forms of laryngeal stenosis, intubation promised to be of the greatest service. In chronic disease intubation was entirely free from danger; many of the difficulties which beset the operation in acute cases, apart from the technique, were primarily due to the highly-inflamed and easily-injured condition of the parts. In chronic cases the laryngeal mucous membrane was usually very tolerant, and cases were recorded in which patients had worn a tube uninterruptedly for ten months. In intubating adults—while on the one hand they could assist the operator more than children would, on the other the distance to the epiglottis was much further, so that the finger was hardly long enough to reach it—something was gained by telling the patient to pull his tongue as far forward as possible.

In a recent number of the "Archives of Pediatrics" Dr. Waxham, of Chicago, gives an analysis of 343 cases intubated. The large number of cases may be taken as a sure indication that every case of croup, as well as every case of diphtheria, is promptly intubated, and the results accordingly are very encouraging. The average number of recoveries is 35·85 per cent. Dr. Pauli * also discusses the question from a large practical experience. Whilst recognising as advantages the easy and rapid execution of the operation, giving instantaneous relief to the breathing, the avoidance of a wound and the danger of infection, the retention of the natural air passages, and the readier consent of the parents to a non-cutting operation, he thinks that they are more than counterbalanced by the great difficulties of the after treatment. These are displacement of the tube from coughing, &c., its blocking up, or its prolapse into the trachea. The process of nourishment is conducted with much trouble, the mucous membrane of the larynx is kept in a state of continuous irritation, and sores frequently arise from the impact of the tube. He declares, further, that pneumonia occurs oftener in those intubated than after tracheotomy. The conclusion he has come to is in acute croupous or diphtheritic stenosis of the larynx, to prefer tracheotomy, and only to use intubation where the assistance is insufficient or the parents decline the cutting operation, and also during a mild epidemic in cases where the throat affection is slight, so that during the feeding of the child the tube may be removed without risk.

A review of these few abstracts may be taken as indicating the average opinion of those who are experienced in the operation. In no surgical procedure are statistics more misleading, since it must be remembered that cases of croup are often intubated when the severe operation of tracheotomy would be out of the question. If tracheotomy is reserved for desperate cases of diphtheria, it is unfair to compare the results with intubation which includes milder cases of croup, unless the difference in the severity of the cases be allowed for.

* *Pract.*, May, 1891.

